# Wie sichern wir in Zukunft die Versorgung von Kindern und Jugendlichen mit psychischen Erkrankungen?

**DOI:** 10.1007/s00103-024-03858-w

**Published:** 2024-03-19

**Authors:** Marcel Romanos, Gundolf Berg, Annegret Brauer, Martin Jung, Marianne Klein, Michael Kölch, Renate Schepker

**Affiliations:** 1 Klinik und Poliklinik für Kinder- und Jugendpsychiatrie, Psychosomatik und Psychotherapie, Universitätsklinikum Würzburg, Margarete-Höppel-Platz, 97080 Würzburg, Bayern Deutschland; 2 Zentrum für ambulante Kinder- und Jugendpsychiatrie und Psychotherapie, Mainz, Rheinland-Pfalz Deutschland; 3 Facharztpraxis für Kinder- und Jugendpsychiatrie, Halle, Sachsen-Anhalt Deutschland; 4 Klinik für Kinder- und Jugendpsychiatrie, Helios Klinikum Schleswig, Schleswig, Schleswig-Holstein Deutschland; 5 Klinik für Kinder- und Jugendpsychiatrie und Psychotherapie, Klinikum am Weissenhof, Weinsberg, Baden-Württemberg Deutschland; 6 Klinik für Psychiatrie, Neurologie, Psychosomatik und Psychotherapie im Kindes- und Jugendalter, Universitätsklinikum Rostock, Rostock, Mecklenburg-Vorpommern Deutschland; 7 Abteilung für Kinder- und Jugendpsychiatrie Weissenau, ZfP Südwürttemberg, Ravensburg, Baden-Württemberg Deutschland

**Keywords:** Kinder- und Jugendpsychiatrie, Psychotherapie, Versorgung, Kinder, Regionalmodell, Child and adolescent psychiatry, Psychotherapy, Care, Children, Regional model

## Abstract

Kinder und Jugendliche mit psychischen Störungen weisen komplexe Bedarfe auf, die regelhaft Leistungen aus mehreren Sozialgesetzbüchern erforderlich machen und innerhalb der medizinischen Versorgung Leistungen aus verschiedenen Sektoren bedingen können. Die steigende Inanspruchnahme, die Zunahme der Schweregrade der Störungsbilder sowie der fachübergreifende Personalmangel belasten die Strukturen der interdisziplinären Versorgungsnetzwerke. Die Folge sind lange Wartezeiten, lange Wegstrecken und regionale Unterversorgung. Das Fachgebiet der Kinder- und Jugendpsychiatrie und -psychotherapie (KJPP) nimmt die zentrale und koordinative Rolle in der kooperativen Versorgung von Kindern und Jugendlichen mit psychischen Erkrankungen ein. Die KJPP weist angesichts der zunehmenden Herausforderungen Reformbedarf auf, welcher sich von dem der somatischen Versorgungsstrukturen, aber ebenso von denen der Psychiatrie und Psychotherapie bzw. der Psychosomatik unterscheidet. Das vorliegende Diskussionspapier beschreibt die Besonderheiten der KJPP-Strukturen, die Reformbedarfe und Lösungsmöglichkeiten durch Abbau von Sektorengrenzen in der Patientenversorgung, in der Weiterbildung sowie im Personaleinsatz, durch Intensivierung der Vernetzung, durch stärkere Nutzung von Telemedizin sowie durch Aufbau evidenzbasierter Prävention und Früherkennung.

## Abstract (en)

The interdisciplinary care of children and adolescents with mental disorders requires services from various German codes of social law and—within the medical care system—enclosing inpatient and outpatient services. The increasing demand, the increase in severity of disorders, and the general shortage of staff in social services put pressure on the structures of the interdisciplinary service networks resulting in long waiting periods, long distances, and regionally insufficient care. The medical field of child and adolescent psychiatry and psychotherapy (CAPP) plays the central and coordinating role within the cooperative care for children and adolescents with mental disorders. The CAPP is in clear need of reforms; however, these are markedly different from the reform needs of the German somatic medical care system and differ substantially from those of the (adult) psychiatry, psychotherapy, and psychosomatics disciplines. This discussion paper describes the reform requirements, the specifics of the CAPP structures, and suggestions to overcome sectors of service provision, enhance networking, intensify telemedicine, and develop evidence-based prevention and early recognition of child mental disorders.

## Status quo und Besonderheiten der kinder- und jugendpsychiatrischen Versorgung in Deutschland

### Einleitung

Psychische Erkrankungen beginnen früh in Kindheit und Jugend und weisen lebenslange Verläufe auf. Bereits vor der COVID-19-Pandemie lagen bei jedem 5. Kind in Deutschland psychische Belastungen vor. Während der Pandemie nahmen diese noch weiter zu und kehren bis heute nur teilweise auf das vorpandemische Niveau zurück [1]. Psychische Erkrankungen bei Kindern und Jugendlichen im Alter von 10–17 Jahren waren im Jahr 2021 die häufigsten Ursachen für Krankenhausbehandlungen [2]. Die Zunahme der Inanspruchnahme ist durch verschiedene Entwicklungen bedingt. Internationale Analysen zur COVID-19-Pandemie legen nahe, dass die absolute Zahl an psychischen Erkrankungen bei Kindern und Jugendlichen tatsächlich zugenommen hat [3], andererseits können eine gestiegene Früherkennung, zunehmende Entstigmatisierung und eine verbesserte Versorgungssituation diese Entwicklung befördern.

Eine Vielzahl von Herausforderungen hat sich in Deutschland für die Versorgungssysteme aufgetan, die anstehende Krankenhausreform ist dabei ein Zeichen des aktuellen Handlungsdrucks. Auch für die „Psych-Fächer“, welche die Pflichtversorgung von Menschen mit psychischen Erkrankungen gewährleisten und die Sicherstellung der Versorgung verantworten, besteht Reformbedarf [4–8]. Dabei weist die kinder- und jugendpsychiatrische Versorgung eine Reihe von Besonderheiten auf, die eine separate Betrachtung erforderlich machen.

Die Versorgung von Kindern und Jugendlichen mit psychischen Erkrankungen erfordert ein interdisziplinäres Versorgungsnetzwerk, welches die Breite der Bedarfe von niederschwelligen Beratungsangeboten über ambulante Diagnostik und Therapie bis hin zu hochintensiver stationärer Akutversorgung abbildet. Die erforderlichen Leistungen umfassen mehrere Sozialgesetzbücher und Sektoren, sodass sich an den Grenzen der jeweiligen Systeme regelhaft Versorgungsdefizite und „Verschiebebahnhöfe“ auftun [4]. Die ambulanten, teilstationären und stationären Strukturen des medizinischen Fachgebiets der Kinder- und Jugendpsychiatrie und -psychotherapie (KJPP) nehmen die zentrale und koordinative Rolle in der Versorgung von Kindern und Jugendlichen mit psychischen Erkrankungen ein. Die Versorgung erfolgt kooperativ im Transitionsbereich zum Erwachsenenalter mit der Psychiatrie und Psychotherapie, im Bereich psychosomatischer Erkrankungen mit der Kinderheilkunde und im Bereich der Psychotherapie mit den Kinder- und Jugendlichen-Psychotherapeut:innen und wird ergänzt durch schulische Hilfen sowie die Jugend- und Eingliederungshilfe.

### Stationäre Versorgungsstrukturen

Seit den 1990er-Jahren wurden Betten in der KJPP erheblich abgebaut, nahmen jedoch zuletzt wieder leicht zu auf aktuell 6700 Betten sowie 4000 tagesklinische Behandlungsplätze [9]. Die abgeleitete Bettenmessziffer (BMZ; Betten pro 10.000 EW unter 18 Jahren) schwankt zwischen den Bundesländern erheblich: Bayern: 3,6; Sachsen-Anhalt: 11,0 (eigene Berechnungen nach [9]). Große bzw. bevölkerungsreiche Bundesländer (BY, BW, NRW, Berlin) weisen vergleichsweise niedrige Bettenmessziffern auf. Die erforderliche Anzahl von stationären Behandlungsplätzen für eine Region ist u. a. abhängig von der ambulanten Versorgungssituation sowie dem Ausbau der Kinder- und Jugendhilfe im Versorgungsgebiet. Die Netzwerkbildung mit der Kinder- und Jugendhilfe ist essenziell, zumal mehr als 70 % aller Patient:innen, die stationär behandelt werden, auch Maßnahmen der Kinder- und Jugendhilfe erhalten [10]. Zudem ist das Auftreten psychischer Störungen u. a. von sozialen Faktoren abhängig, d. h., Regionen mit höherer Armutsquote können auch höhere Bedarfe haben. Schließlich spielen geografische Faktoren eine Rolle: In urbanen Verdichtungsräumen können beispielsweise aufgrund guter Erreichbarkeit tagesklinische Behandlungsplätze vollstationäre Plätze eher ersetzen als in ländlichen Regionen.

Die BMZ im stationären KJPP-Bereich sind deutlich geringer als in der Erwachsenenpsychiatrie, welche wiederum deutlich höhere Fallzahlen versorgt. In der Folge sind die Versorgungs- und Planungsgebiete der KJPP um etwa den Faktor 3 größer, sodass weniger Kliniken für deutlich größere Regionen Versorgungsverantwortung tragen. Mit wenigen Ausnahmen haben alle universitären wie nicht-universitären KJPP-Fachkliniken in Deutschland Versorgungspflicht bzw. übernehmen einen Sicherstellungsauftrag. Im Gegensatz zu somatischen Kliniken ist daher ein „Abmelden“ eines Krankenhauses von der Versorgung nicht möglich. Diese Besonderheit der psychiatrischen Versorgungsstruktur wurde auch von der Regierungskommission in ihrer Stellungnahme als vorbildhaft bewertet. Eine weitere Aggregierung von Leistungen durch Klinikschließungen und Zentralisierungen, wie sie im Rahmen der anstehenden Krankenhausreform [11] für somatische Kliniken bevorsteht, ist daher unplausibel und würde unmittelbar zu regional katastrophalen Versorgungseinbrüchen führen.

### Ambulante Versorgungsstrukturen

Der ambulante Sektor weist große Versorgungsunterschiede auf. In ländlich geprägten Regionen finden sich schwächere Versorgungsstrukturen mit langen Fahr- und Wartezeiten, ebenso aber auch in den benachteiligten Stadtteilen großer Städte. Der größte Anteil der betroffenen Kinder und Jugendlichen wird ambulant in kinder- und jugendpsychiatrischen Facharztpraxen versorgt, welche nach der Sozialpsychiatrie-Vereinbarung (SPV; [12]) nach § 85 Abs. 2 Satz 4 und § 43a SGB V arbeiten. Das SPV-Modell fördert die qualifizierte interdisziplinäre sozialpsychiatrische Behandlung in der ambulanten vertragsärztlichen Versorgung. Durch ein interdisziplinäres Team werden umfassende ambulante Angebote durch mehrere Berufsgruppen geboten, wodurch stationäre Behandlungen reduziert werden sollen. Die SPV wird ergänzt werden durch die anstehende „Richtlinie über die berufsgruppenübergreifende, koordinierte und strukturierte Versorgung, insbesondere für schwer psychisch kranke Kinder und Jugendliche mit komplexem psychiatrischen oder psychotherapeutischen Behandlungsbedarf“ (KJ-KSVPsych RL) des Gemeinsamen Bundesausschusses (G-BA) nach § 92 Abs. 6b, SGB V. Dieser Ansatz soll die regionale Vernetzung zwischen Leistungserbringern intensivieren, um die Behandlungsqualität schwer und chronisch kranker Kinder und Jugendlicher zu verbessern.

Zusätzlich sind im ambulanten Bereich ca. 7000 Kinder- und Jugendlichen-Psychotherapeut:innen tätig [13]. In vielen schwach versorgten Regionen unterstützen psychiatrische Institutsambulanzen die ambulante Regelversorgung. Insbesondere im Bereich der Entwicklungsstörungen und bei somatischer Komorbidität erfolgt ein relevanter Anteil der Versorgung in sozialpädiatrischen Zentren (SPZ). Insgesamt sind Strategien für eine Ausweitung der Ambulantisierung bereits vonseiten des ambulanten Sektors gut konzeptualisiert, jedoch keineswegs flächendeckend ausreichend umgesetzt, insbesondere aufgrund regionaler Unterschiede vom bis zu 6‑Fachen der Facharztdichte. Insuffiziente strukturelle Verzahnungen zwischen dem ambulanten und stationären Sektor führen darüber hinaus zu einem Informationsverlust, Mehrfachuntersuchungen sowie Versorgungsabbrüchen der besonders schwer betroffenen Patient:innen. Zu einer umfassenden Darstellung der KJPP-Versorgung siehe [14].

### Hohe Koordinierungsaufwände

Grundsätzlich bestehen in der KJPP trialogische Behandlungssettings durch den notwendigen Einbezug der Familien. Die dadurch erhöhten fachlichen, zeitlichen und administrativen Aufwände in der Behandlung von Kindern und Jugendlichen sind nicht reduzierbar, sie stellen wesentliche Qualitätsmerkmale des Faches dar. Nur dadurch kann es gelingen, die Familien als Ressource zu nutzen und soziale Teilhabe im möglichst ursprünglichen Umfeld zu ermöglichen. Vor dem Hintergrund der o. g. Schnittstellenprobleme zwischen unterschiedlichen Sozialleistungen und Hilfeerbringern ist die Sicherung von parallelen Leistungen, z. B. der Jugendhilfe, unabdingbar. Regional wurden vielerorts Kooperationsstrukturen im gemeindepsychiatrischen Sinne mit Schulen, Jugendämtern und Hilfesystemen zur Sicherung von Integration und Teilhabe aufgebaut. Insofern bestehen Konzepte und umfangreiche Praxiserfahrungen zur SGB- bzw. sektorübergreifenden Versorgung, jedoch sind kinder- und familienzentrierte Anpassungen der Regularien erforderlich, um diese Konzepte flächendeckend umsetzen zu können. Die beschriebenen Versorgungsstrukturen werden aktuell durch eine Vielzahl von komplexen Herausforderungen einem bundesweiten Stresstest unterzogen und in der aktuellen Entwicklung wird ein grundlegender Reformbedarf vonseiten der Krankenkassen, der Regierung, der Krankenhausträger sowie der Fachgesellschaften gleichermaßen konstatiert. Unklar bleibt, wie diese Reform zu gestalten sei, allerdings könnte aus unserer Sicht über einige Kernelemente Konsens erreicht werden. Das vorliegende Thesenpapier möchte eine Diskussionsgrundlage für den möglichen Weg einer Neuordnung der Versorgung liefern und verschiedene Ansatzpunkte aus fachlicher Sicht strukturiert darstellen, unter Einbezug der Perspektiven des ambulanten sowie stationären Sektors.

## Herausforderungen und Reformbedarf

### Wachsende Inanspruchnahme

Innerhalb von 2 Jahrzehnten hat sich die Zahl der berufstätigen Fachärzt:innen für Kinder- und Jugendpsychiatrie und -psychotherapie von 1026 (im Jahr 2000) auf 2776 (im Jahr 2022) nahezu verdreifacht [15], wodurch bestehende Versorgungsdefizite in der Fläche teils abgemildert werden konnten. Jedoch hat die Coronapandemie die bereits zuvor steigende Inanspruchnahme und Schweregradzunahme psychischer Störungen bei Kindern und Jugendlichen weiter akzeleriert und Stärken wie auch strukturelle Defizite der Versorgungsysteme offenbart [16, 17]. Prognosen gehen von einer weiteren Steigerung kassenärztlicher Leistungen im Bereich Psychotherapie bis 2030 um 23 % aus. Gleichzeitig wird eine weitere Verdichtung der Inanspruchnahme im urbanen Raum gegenüber ländlichen Regionen erwartet [18]. Die aktuellen übergreifenden krisenhaften Entwicklungen stellen ernsthafte Bedrohungen für die Sicherstellung der Versorgung von Kindern und Jugendlichen mit psychischen Erkrankungen in Deutschland dar [19].

### Interdisziplinärer Fachkräftemangel

Der inzwischen disziplin- und fachübergreifende Fachkräftemangel bedroht nicht nur aktuell die Versorgungssicherung, sondern wird noch dauerhaft verschärft durch einen zunehmenden Nachwuchsmangel im therapeutischen, pflegerischen und erzieherischen Bereich. Die Folge sind fehlende Nachfolger:innen für niedergelassene Praxisinhaber:innen, nicht besetzte Arztstellen in den Kliniken, sowohl bei den Berufsanfänger:innen als auch in Leitungspositionen, sowie fehlendes Fachpersonal im therapeutischen Bereich und im Bereich des Pflege- und Erziehungsdienstes. Diese Entwicklung wird verstärkt durch eine zunehmende Inanspruchnahme von Teilzeitarbeit in allen Bereichen, insbesondere aber durch die Abwerbung von Fachpersonal der KJPP durch psychosomatische Kliniken, welche außerhalb der Akutversorgung mit kinder- und jugendpsychiatrischen Konzepten und Personal nur selektierte Patient:innengruppen versorgen. Der medizinische Personalmangel wurde über Jahrzehnte aber auch dadurch befördert, dass die KJPP trotz vielfältiger Bemühungen kein curriculares Approbationsfach ist und erst mit der aktuell avisierten Neuordnung der medizinischen Ausbildung die Vernachlässigung von psychischen Erkrankungen in der Medizin reduziert werden soll [20].

Aber auch in den regionalen Netzwerken fehlt zunehmend pädagogisches und therapeutisches Fachpersonal in ambulanten sowie stationären Jugendhilfestrukturen, sodass die über die Jahre aufgebauten interdisziplinären Versorgungsketten und -netzwerke zu versagen drohen. Diese Personalentwicklung wirft zunehmend die Frage auf, welche Zersplitterung der Versorgungslandschaft noch tragbar ist und an welcher Stelle und in welcher Situation eine Ressourcenallokation erforderlich wird.

### Mindestvorgaben für Personaleinsatz

Mit der Einführung der Personalausstattung Psychiatrie und Psychosomatik-Richtlinie (PPP-RL) werden Mindestvorgaben für den Personaleinsatz in den sogenannten Psych-Fächern definiert, die primär der Qualitätssicherung dienen sollen. Die PPP-RL hat jedoch nicht nur durch neue Dokumentations- und Nachweispflichten den bürokratischen Aufwand weiter erhöht, sondern wurde von Anfang an als Budgetfindungsinstrument von den Krankenkassen fehlinterpretiert [21]. Vielmehr stellt die PPP-RL die Untergrenze der berufsgruppenbezogenen Personalausstattung fest, unterhalb derer Pflege und psychiatrische Versorgung nicht mehr sicher zu gewährleisten sind. Heftig umstritten waren auch die unverhältnismäßig hohen Strafzahlungen der Krankenhäuser bei Unterschreitung der Mindesterfüllung der PPP-RL, welche Krankenhäuser in eine existenziell bedrohliche Spirale geschickt hätte. Im Rahmen des aktuellen Aussetzens und der Ankündigung der Überarbeitung der Sanktionsmechanismen besteht die Chance für eine neue Definition der Personalvorgaben, welche die stationäre Versorgung psychisch kranker Kinder und Jugendlicher langfristig sichert, statt sie zu gefährden.

### Sektorengrenzen

Die Forderungen nach einer stärkeren Ambulantisierung als Ausweg aus den zunehmenden Kosten und dem wachsenden Personalmangel implizieren die Notwendigkeit für intensive stationsersetzende Versorgungskonzepte. Diese werden teils durch bestehende Sektorengrenzen behindert, teils durch die unflexible Refinanzierungssystematik im stationären Sektor. Innovative Modellprojekte nach § 64b SGB V wurden zwar vorrangig im erwachsenenpsychiatrischen Bereich durchgeführt [22], die wenigen kinder- und jugendpsychiatrischen Modelle adressierten jedoch sämtlich Sektorengrenzen und eine Flexibilisierung der Ressourcenverwendung innerhalb des ambulanten bzw. des stationären Sektors, beispielsweise durch neue intensive ambulante Konzepte oder stationsersetzende Behandlungsangebote für ansonsten stationäre oder teilstationäre Patient:innen. Erfolgreich durchgeführte Modellprojekte stellen gute Blaupausen für eine flächendeckende Neuordnung der Versorgung dar.

## Lösungsansätze zur Sicherung der Versorgung

### Mehr Durchlässigkeit und Transparenz im ambulanten und stationären Sektor

Die Schnittstelle zwischen den Sektoren ist ein notorisches Problemkind der medizinischen Versorgung angesichts von Wartezeiten, Informationsverlusten und Behandlungsabbrüchen. Zu fordern ist eine wechselseitige Verfügbarmachung von Behandlungsplätzen zur nahtlosen Weiterversorgung (ambulant zu stationär, stationär zu ambulant) mit dem Ziel, Wartezeiten zwischen den Behandlungssettings zu minimieren, Aggravierungen bzw. Rückfälle zu reduzieren und Behandlungsabbrüche zu vermeiden. Diese Forderung impliziert eine konsequente fachliche Abstimmung zwischen Niedergelassenen und Klinik hinsichtlich der Indikationen für Aufnahme und Weiterbehandlung sowie Definition von Behandlungszielen und deren wechselseitige Akzeptanz. Die Refinanzierung der Schnittstellenarbeit ist aktuell weder im stationären noch im ambulanten Bereich suffizient abgebildet. Letztlich werden schwer erkrankte Patient:innen einer kooperativen Versorgung bedürfen, wofür bisherige Ausschlussgründe wegfallen müssen, z. B. parallele Leistungserbringung durch ambulante und stationäre Behandler zur Sicherung nahtloser Übergänge. Gleiches gilt bei Patient:innengruppen, die parallel einen somatischen und psychiatrischen Behandlungsbedarf haben und für welche eine parallele ambulante Behandlung in sozialpädiatrischen Zentren und Institutsambulanzen bislang ausgeschlossen ist. Im Gegenzug für die Aufhebung der Leistungsausschlüsse wird eine Definition notwendig werden, wodurch sich diese Patient:innen auszeichnen, um einer ungebremsten Mengenausweitung entgegenzutreten. Bisherige Versuche, aus Routinedaten eine Schwergraddefinition zu entwickeln, sind daran gescheitert, dass sich aus einer reinen Diagnose nicht die Bedarfe objektiv ableiten lassen. In der Kinder- und Jugendpsychiatrie und -psychotherapie hat sich das multiaxiale Klassifikationsschema (MAS; [23]) bewährt, welches auf 6 Achsen neben den psychiatrischen Diagnosen die somatischen Erkrankungen, Entwicklungsstörungen, das Intelligenzniveau, psychosoziale Faktoren sowie eine Globalbeurteilung des Funktionsniveaus codiert. Dieses System kann störungs- und sektorübergreifend die Basis für eine objektive und transparente Bedarfsermittlung sein. Auch die Internationale Klassifikation der Funktionsfähigkeit, Behinderung und Gesundheit (ICF; [24]) stellt ein interdisziplinär anwendbares Instrument dar, um SGB V-übergreifende Bedarfe zu objektivieren und effektiv zu planen (vgl. folgendes Unterkapitel).

Neben der sektorübergreifenden Kooperation zur Sicherung der Versorgungsbedarfe sind in den jeweiligen Regionen aus Ressourcen– sowie aus Qualifikationsgründen durchaus Modelle denkbar, die spezifische und spezialisierte Angebote (z. B. Diagnostik von Autismus-Spektrum-Störungen, spezielle Therapieangebote bei Intelligenzminderung) nicht von allen Leistungsanbietern vorsehen, sondern auf spezifische Anbieter unabhängig von den Sektorengrenzen verteilt werden. Dennoch muss eine bezugstherapeutische Begleitung möglich sein, die auch die Angebotsnutzung steuert. So könnte eine verbesserte Durchlässigkeit die Qualität der Versorgung erhöhen und dennoch Ressourcen sparen.

### Intensivierung der lokalen Netzwerkbildung

Die lokale Vernetzung innerhalb der KJPP zwischen ambulantem und stationärem Sektor ist von grundlegender Bedeutung, jedoch nur ein Baustein für eine gelingende Versorgung. Die kooperative Verzahnung mit den Kinder- und Jugendlichen-Psychotherapeut:innen ist wesentlich für die psychotherapeutische Versorgung vor, aber insbesondere auch im Anschluss an eine stationäre Krisenintervention oder Behandlung. Kooperative sowie komplementäre Modelle mit den Nachbardisziplinen der Kinderheilkunde (Psychosomatik, Sozialpädiatrie) und Psychiatrie (Transition) versorgen Gruppen mit spezifischen Bedarfen. Psychische Störungen bedingen jedoch regelhaft komplexe Bedarfe, welche neben SGB V auch SGB VIII und weitere Sozialleistungen umfassen. Bei einem Großteil der stationären KJPP-Patient:innen sind Jugendhilfemaßnahmen parallel initiiert oder indiziert und umgekehrt haben viele Patient:innen in der stationären Jugendhilfe behandlungsbedürftige psychische Störungen [10].

Viele regionale Netzwerkbildungen funktionieren zum Teil schon gut und können damit als Praxismodelle für regionale Adaptationen zur Verfügung stehen. Praxisbeispiele finden sich hier: [25]. Ein wesentlicher Aspekt funktionierender Netzwerke ist die systematische interdisziplinäre Versorgung von Hochrisikogruppen, so z. B. von Kindern und Jugendlichen in der stationären Jugendhilfe, die durch einen kinder- und jugendpsychiatrischen Konsiliardienst versorgt werden. Wird dieser durch eine Psychiatrische Institutsambulanz (PIA) geleistet, darf das beispielsweise eine fortlaufende psychotherapeutische Richtlinientherapie nicht verunmöglichen. Punktuell existieren Versorgungskonzepte im Bereich psychischer Erkrankungen und Verhaltensstörungen bei komplexen Behinderungen, z. B. durch aufsuchende Konsil- und Liaisondienste in Einrichtungen für Kinder mit Mehrfachbehinderung mit dem Ziel, stationäre Aufenthalte zu verhindern. In der Regel sind die erforderliche Vernetzungsarbeit und erhöhten Aufwände bislang nicht refinanziert. Kontakte zur Arbeitsagentur, Berufsbildungseinrichtungen, Schulämtern und Schulen und der künftig mit allen Inklusionsaufgaben beauftragten Jugendhilfe müssen hierbei gleichermaßen mitgedacht werden.

### Implementierung von Regionalmodellen

Die Modellprojekte nach § 64b beziehen sich häufig auf die Schaffung von sogenannten Regionalmodellen, in welchen ein Regionalbudget für die Versorgung zur Verfügung steht und bedarfsangemessen Ressourcen alloziert werden. In den ersten Versuchen gehen die Modelle grundsätzlich von den Krankenhausstrukturen einer Region aus, die in der Kinderpsychiatrie dünn gesät sind, weshalb die Modelle bis dato nur qualitativ auswertbar gewesen sind [26]. Jedoch sind bereits Weiterentwicklungen dieses Ansatzes angedacht mit einer übergreifenden Regionalplanung von ambulantem und stationärem Sektor. Sogar der Einbezug von Jugendhilfe und Eingliederungshilfe sowie aller weiteren Sozialleistungen in ein umfassendes regionales Budget wurde skizziert [5].

Obgleich dieser Vision eine Vielzahl von praktischen und rechtlichen Hindernissen entgegenstehen, sehen wir diese Konzeptualisierung als richtigen Schritt an. Voraussetzung wäre eine „regionale Pflichtversorgung“ aller Leistungserbringer für die minderjährigen Bewohner:innen der Raumschaft. Die Verpflichtung der Sicherstellung der Versorgung würde damit von den stationären Versorgungsstrukturen auf das regionale Netzwerk mit dem gesamten Spektrum der Versorgungsintensitäten übergehen. Eine solche Weiterentwicklung der Versorgungsstrukturen macht eine funktionierende Feststellung der Bedarfe erforderlich, die eine gemeinsame Falldefinition und Zuweisung in das jeweilige Angebot impliziert. Angesichts der rasanten Entwicklung der Leistungsfähigkeit künstlicher Intelligenz möchten wir nicht ausschließen, dass auch diese in einer solchen Konzeption einen umschriebenen Stellenwert bekommen kann.

Das erwünschte Ergebnis dieses Ansatzes in einer Region ist eine bedarfsabhängige stratifizierte Versorgung (*Stratified Care*) verbunden mit einer effizienten Weitervermittlung der Patient:innen zwischen den verschiedenen Leistungserbringern bei Änderung der Bedarfe im Sinne eines gestuften Konzepts (*Stepped Care*). Das Konzept müsste selbstverständlich durch eine sich mitentwickelnde regionale Sozialplanung der Angebotsstrukturen begleitet werden und würde letztlich auch kommunale und föderale (schulische/berufliche) Ressourcen, aber auch überwiegend überregional arbeitende Rehabilitationsstrukturen berühren.

### Verantwortungsübernahme, Flexibilisierung und Ressourcenallokation

Der Funktionalität eines wie auch immer definierten regionalen interdisziplinären Netzwerks ist eine natürliche Grenze gesetzt durch die Verfügbarkeit von ausreichend Personalressourcen im jetzigen ambulanten sowie stationären Sektor. Beide benötigen wiederum für eine nachhaltige Effizienz ihrer Leistungserbringung eine funktionierende Jugendhilfe zur komplementären Deckung der komplexen Bedarfe. Zum wesentlichen Merkmal eines Regionalmodells – d. h. der geteilten und damit gemeinsamen Verantwortung für die Versorgung aller Hilfeempfänger in einer Region – gehört auch, dass das Netzwerk bei personellen Defiziten innerhalb der Systeme adaptiv auf die veränderte Angebotssituation reagieren kann. Hierzu müssen Möglichkeiten geschaffen werden, innovative Konzepte rasch umsetzen zu können, beispielsweise die Ausweitung ambulanter Leistungen anstelle stationärer Behandlung oder die Schaffung von Belegarztsystemen bei erhöhtem Aufkommen stationärer Indikationen. Gemeinsame Weiterbildungsbefugnisse der ambulanten und stationären Leistungserbringer wären eine logische Folge. Schließlich müssen auch fachärztliche und fachpsychotherapeutische Personalressourcen adaptiv alloziert werden können, um die Gesamtversorgung in der Region sicherstellen zu können. In dieser Konzeption müssen auch Personalressourcen hochselektiver Versorgungsstrukturen, wie sie aktuell z. B. in psychosomatischen Kliniken gebunden werden, in der Breite der Bedarfe eingesetzt werden.

Zusätzlich werden innovative ambulante Angebote, gerade in unterversorgten Regionen, entwickelt werden. Aus besser versorgten Regionen heraus müssen Angebote von Kliniken sowie Niedergelassenen entwickelt werden, wie z. B. tageweise Sprechstunden in der Peripherie. Kommunen können einen Beitrag leisten, indem sie Räumlichkeiten zur Verfügung stellen. Zur Versorgung ländlicher Gebiete hat sich bereits während der Coronapandemie die Telemedizin als leistungsfähiges Konzept in der Praxis bewährt. Videosprechstunden ermöglichen ressourcensparende, flexiblere und höherfrequente Kontakte, wegfallende Fahrtzeiten und raschere Interventionen oder gar Verhinderung krisenhafter Verläufe. Obgleich persönliche Beziehungen bei behandlungsbedürftigen Kindern und Jugendlichen durch digitale Anwendungen („Therapie-Apps“) kaum ersetzt werden können, so können die Therapien jedoch durch die Digitalisierung unterstützt werden.

Wir gehen davon aus, dass bei einer individualisierten bedarfsadaptierten Ressourcenallokation erhebliche Einsparungen möglich sind. Teure vollstationäre Zeiten können verkürzt und flexibilisiert werden durch stationsäquivalente, teilstationäre und ambulante Angebote. Auch die Übergänge in klinikfernere Angebote und umgekehrt schnellere Zugänge zu z. B. Kriseninterventionen vom ambulanten Sektor aus können durch Aufheben der Schnittstellenprobleme ebenfalls erhebliche Doppelarbeit und damit Kapazitäten einsparen [27]. Die Erfahrung, in Betreuungskontinuität arbeiten zu können, macht die Tätigkeit für alle Berufsgruppen attraktiver und dürfte zur Personalgewinnung und Personalbindung beitragen. Daraus resultierende Anforderungen auf die Aus- und Weiterbildung sollten allerdings unmittelbar umzusetzen sein.

### Prävention und Früherkennung

Um den erheblichen Herausforderungen der Versorgungsstrukturen angemessen begegnen zu können, ist es erforderlich, der kontinuierlichen Zunahme der Inanspruchnahme durch effektive präventive Maßnahmen zu begegnen. Dazu sind wirksame, evidenzbasierte und kosteneffektive Konzepte erforderlich, die trotz des exzessiven Vorhandenseins von Präventionsprogrammen angesichts unzureichender wissenschaftlicher Evaluation weitestgehend fehlen. Das Präventionsgesetz hat erste Weichenstellungen vorgenommen, jedoch werden die den Krankenkassen zur Verfügung stehenden Mittel im großen Umfang mehr zur Imagepflege denn zur tatsächlichen Entwicklung wirksamer Prävention verwendet. Es bedarf einer transparenten Verwendung dieser Mittel für die Entwicklung evidenzbasierter Programme sowie Evaluation existierender Ansätze verbunden mit konsequenten bundesweiten Disseminationsstrukturen für positiv evaluierte Konzepte. In diesem Kontext kommt auch existierenden Strukturen der Früherkennung und Prävention besondere Bedeutung zu, die es weiterzuentwickeln und in ihrer Wirksamkeit zu steigern gilt.

Der Öffentliche Gesundheitsdienst (ÖGD) weist im Grundsatz exzellente Voraussetzungen auf, um frühzeitig Risikokonstellationen und frühe psychische Gesundheitseinschränkungen zu detektieren, jedoch fehlen zur Erfüllung dieser Funktion wesentliche Verzahnungen mit Leistungen anderer Sozialgesetzbücher. Oftmals fehlen dem ÖGD notwendige Befugnisse und Ressourcen, um die konsequente Umsetzung seiner Empfehlungen zu monitoren. So könnte eine Aufwertung der Schuleingangsuntersuchung zur konsekutiven Überführung von Kindern mit früher Ausprägung psychischer Störungen in niederschwellige Leistungen des SGB V gereichen [28]. Eine systematische Ausweitung auf Kindergartenuntersuchungen steht aus [29]. Oftmals fehlen dem ÖGD notwendige Befugnisse und Ressourcen, um die konsequente Umsetzung seiner Empfehlungen zu monitoren [30], ebenso fehlt der Einbezug weiterer Gesundheitsberufe.

## Fazit

Eine steigende Inanspruchnahme, Personalmangel und eine Zersplitterung der Systeme bedrohen die Versorgung von Kindern und Jugendlichen mit psychischen Erkrankungen. Die interdisziplinären Versorgungsstrukturen müssen sich weiterentwickeln, um auch zukünftig bedarfsangemessene Hilfe leisten zu können. Die Kinder- und Jugendpsychiatrie und -psychotherapie hat als zentraler Akteur eine besondere Verantwortung, die erforderlichen Entwicklungen zu konzeptualisieren und im interdisziplinären Verbund zu etablieren. Die erprobten Regionalmodelle bieten dafür eine gute Grundlage für stratifizierte und gestufte Versorgungsangebote. Die regionalen Verbundkonzepte müssen jedoch noch weiter konkretisiert und regional adaptiert werden, um eine tatsächliche gemeinsame Verantwortungsübernahme für das gesamte Spektrum der Bedarfe realisieren zu können.
